# Best Practices in Structural Neuroimaging of Neurodevelopmental Disorders

**DOI:** 10.1007/s11065-021-09496-2

**Published:** 2021-04-24

**Authors:** Lea L. Backhausen, Megan M. Herting, Christian K. Tamnes, Nora C. Vetter

**Affiliations:** 1 Department of Child and Adolescent Psychiatry, Faculty of Medicine of the Technische Universitaet Dresden, Dresden, Germany; 2 Department of Preventive Medicine, Keck School of Medicine, University of Southern California, Los Angeles, CA USA; 3 PROMENTA Research Center, Department of Psychology, University of Oslo, Oslo, Norway; 4 NORMENT, Institute of Clinical Medicine, University of Oslo, Oslo, Norway; 5 Department of Psychiatric Research, Diakonhjemmet Hospital, Oslo, Norway

**Keywords:** Neurodevelopmental disorders, Children, Structural MRI, Study design, Quality control, FreeSurfer

## Abstract

Structural magnetic resonance imaging (sMRI) offers immense potential for increasing our understanding of how anatomical brain development relates to clinical symptoms and functioning in neurodevelopmental disorders. Clinical developmental sMRI may help identify neurobiological risk factors or markers that may ultimately assist in diagnosis and treatment. However, researchers and clinicians aiming to conduct sMRI studies of neurodevelopmental disorders face several methodological challenges. This review offers hands-on guidelines for clinical developmental sMRI. First, we present brain morphometry metrics and review evidence on typical developmental trajectories throughout adolescence, together with atypical trajectories in selected neurodevelopmental disorders. Next, we discuss challenges and good scientific practices in study design, image acquisition and analysis, and recent options to implement quality control. Finally, we discuss choices related to statistical analysis and interpretation of results. We call for greater completeness and transparency in the reporting of methods to advance understanding of structural brain alterations in neurodevelopmental disorders.

## Introduction

Knowledge about the prevalence, clinical diagnosis and treatment of frequent neurodevelopmental disorders has increased considerably over the past two decades. These comprise, for example, attention deficit hyperactivity disorder (ADHD; prevalence approx. 6 %; Barkley, 2014), conduct disorder (CD; prevalence approx. 3 %; Canino et al., 2010), and oppositional defiant disorder (ODD; prevalence approx. 3 %; Canino et al., 2010). These externalizing disorders often persist into adulthood, impacting quality of life dramatically (Kessler et al., 2005). To prevent worsening of symptoms and life-long suffering from these disorders, it is crucial to investigate and understand their complex neurobiology, which may enable early diagnosis, prevention, prognosis, and treatment during childhood and adolescence.

Certainly, several challenges remain in improving prevention and treatment of these disorders. First, the etiology of neurodevelopmental disorders is still largely unknown, but likely comprises a mix of factors ranging from genetics (Blesson & Cohen, 2020) to epigenetics and environmental influences (Nigg, 2012). Moreover, to date, neurodevelopmental disorders are phenomenologically defined and diagnosed by clinical observations and reports from the child and caregivers. However, classification into clinical categories may not match individual circumstances, problems, and needs (Hyman, 2010). Consequently, psychopharmacological and psychotherapeutic treatments do not help all patients. Therefore, researchers and clinicians should consider neurobiological processes, together with psychological mechanisms, to gain a more holistic picture of neurodevelopmental disorders (see also Research Domain Criteria; RDoC, Sanislow et al., 2019). One invaluable tool that has the potential to identify neurobiological markers, and inform diagnosis and therapy is structural magnetic resonance imaging (sMRI). SMRI allows researchers and clinicians to quantify aspects of brain morphometry, such as the size and shape of specific brain structures and regions. By showing how structural brain maturation in neurodevelopmental disorders deviates from typical developmental trajectories, sMRI may advance understanding of disorder etiology and ontogeny. Beyond increasing knowledge about the neurobiological underpinnings of particular neurodevelopment disorders, a possible research and clinical application could be to directly compare and contrast brain developmental patterns across disorders and subgroups (see Opel et al., 2020), and address questions about transdiagnostic versus disorder-specific developmental abnormalities. Further, it has been suggested that sMRI one day might even provide brain growth charts, similar to existing charts for height, weight and head circumference, which may be used at the individual level to help identify developing disorders early on. Pioneering studies in this field are emerging (Marquand et al., 2019). For example, Dong et al., (2020) analyzed data from two accelerated longitudinal cohorts from China and the United States, including in total 590 typically developing children with 864 scans, and generated brain volumetric growth charts. Importantly, certain growth differences between the two cohorts were observed. Two possible causes of such differences are methodological differences between sites and ethnicity differences, with both of these possibilities requiring increased attention in future endeavors to develop brain growth charts. Moreover, in this context where individual brain developmental differences, including infrequent atypically developmental trajectories, are of interest, existing studies are still limited in terms of sample size or cross-sectional design. For brain growth charts to be useful, it might also, at least in some situations, be necessary to scan the individual child undergoing clinical examination repeatedly over time. This may not only provide a static comparison with the variation in brain structure across children at the same age, but may also allow mapping her or his brain developmental trajectories, similar to how the body growth charts typically are used.

In order to achieve these goals, the design, implementation, and interpretation of sMRI studies require particular attention. First, theoretical foundations of different brain morphometry metrics need to be considered when formulating hypotheses and choosing data analysis tools. Second, the recruitment of children and adolescents with neurodevelopmental disorders is often challenging. Third, these participants tend to move more during data acquisition, which often produces artifacts in the data. Trying to minimize movement by ensuring compliance, training of study personnel regarding movement, controlling data quality and employing quality control software tools are important steps. Further important challenges comprise the choice of statistical analysis approaches, including selecting appropriate thresholds for multiple comparison correction. In this paper we focus on mid-childhood (5 years) to early adulthood (24 years), as there are almost no longitudinal sMRI data on neurodevelopmental disorders for early childhood due to several methodological challenges. These comprise techniques to manage participant anxiety or excessive movement, technical obstacles like availability of child-appropriate equipment, and special data analysis techniques like the use of pediatric atlases to parcellate brain structure; as discussed in detail by Raschle et al. (2012). Moreover, we focus on brain's gray matter and the semi-automated processing tool FreeSurfer, but refer to other tools where appropriate.

After giving an overview of metrics commonly used to probe brain morphometry, we will review typical neuromaturation from childhood to early adulthood and structural alterations in selected neurodevelopmental disorders, that is, ADHD, CD, and ODD. These frequent externalizing disorders with childhood onset exemplify key challenges in sMRI studies with neurodevelopmental disorders like younger age and hyperactivity in the scanner. For reviews on structural alterations of the less frequent, yet extensively studied autism spectrum disorder, please refer to Bednarz and Kana (2018) and Ecker et al. (2015). Please note that the methodological considerations throughout this paper are not restricted to these disorders but instead apply to the diverse range of neurodevelopmental disorders and studies of typical development.

In this review we provide a hands-on, start-to-finish overview of challenges in clinical neurodevelopmental sMRI research, including suggestions on how to improve practices. We will discuss these challenges following the chronological order of any sMRI study: study design, image acquisition, image processing, quality control of data, and finally, statistical analysis and interpretation.

## Brain Morphometry Metrics

To display brain structure, T1-weighted sequences depict high signal (i.e. lighter areas) for fat content as in white matter, and lower signal (i.e. darker areas) for more water content as in cerebrospinal fluid, skull and gray matter (Westbrook & Talbot, 2018). Gray matter consists of neuron bodies, glial cells, dendrites, blood vessels, extracellular space, unmyelinated and myelinated axons. It is found in the outer layer of the cerebrum (cerebral cortex), as well as in subcortical structures, and in the cerebellar cortex. White matter consists chiefly of long-range myelinated axons and is found in the cerebrum and the cerebellum (Mills & Tamnes, 2014). As the majority of sMRI studies of neurodevelopmental disorders focused on gray matter, this will also be the main focus in this paper.

One established method to characterize the volume and density of gray matter in brain structures is voxel-based morphometry (VBM; Ashburner & Friston, 2000). However, these volume output metrics consist of a mixture of complex underlying effects, which has also lead to criticism (Ashburner, 2009; Davatzikos, 2004). In the following, we will hence focus on surface-based and volume-based analysis in semi-automated tools like FreeSurfer (Fischl, 2012), which allow the individual investigation of several morphometry metrics (see Textbox 1). For a short review of major differences between VBM and surface-based analysis, please see Greve (2011).

### Surface-based and Volume-based Analysis

**Fig. 1:**
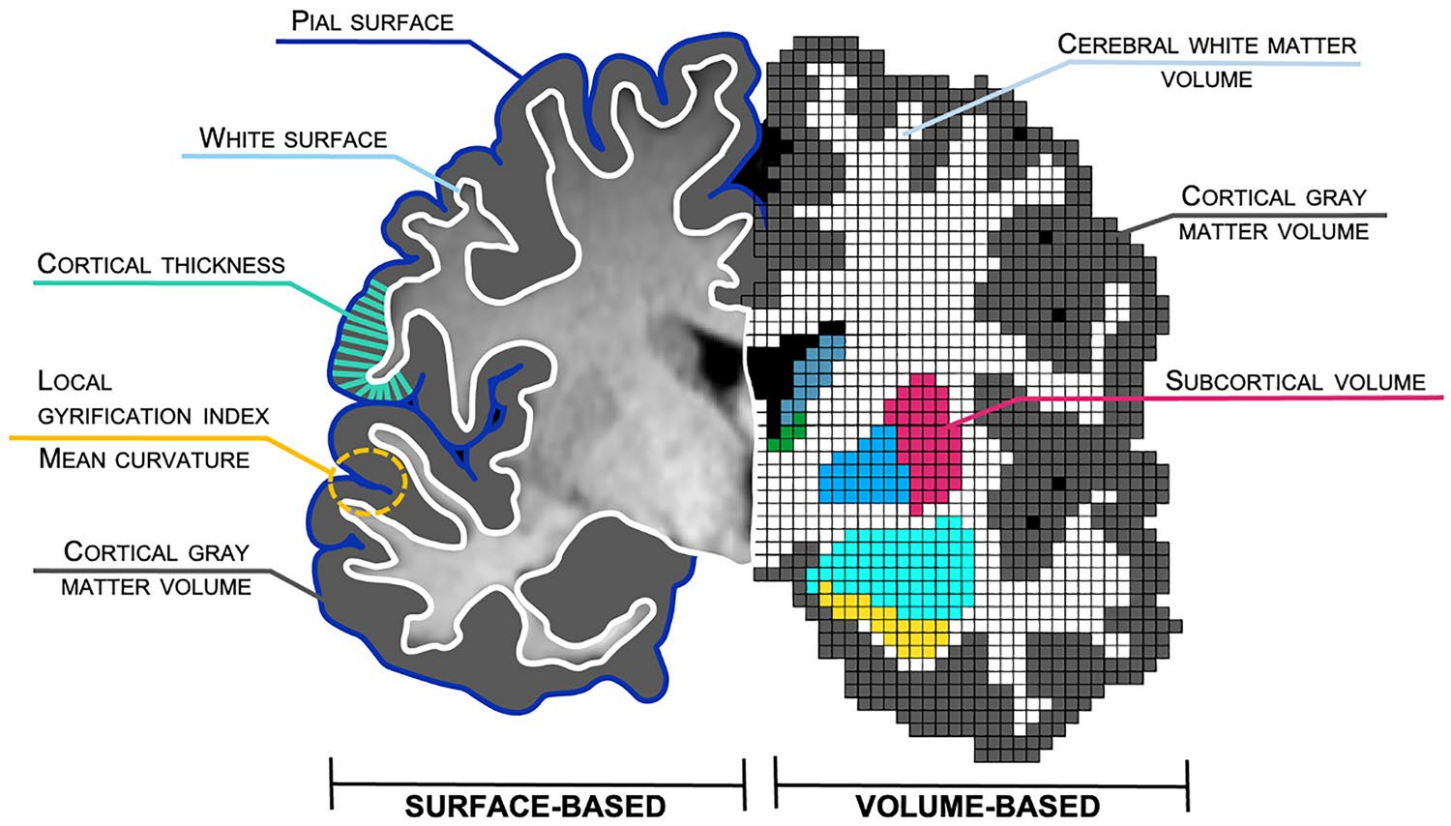
Overview of structural brain metrics. Coronal slice of an individual brain indicating metrics according to the surface-based (left) and volume-based (right) processing streams as implemented in FreeSurfer. Depicted are the subcortical structures nucleus caudatus (blue-gray), thalamus (green), putamen (magenta), globus pallidus (deep sky blue), amygdala (cyan), and hippocampus (yellow). Black represents cerebrospinal fluid. Corpus callosum and ventricles are not labeled. For illustration purposes, the graphic and scaling was simplified and does not claim anatomical correctness. Figure courtesy of Anna Backhausen

FreeSurfer (Fischl, 2012) is widely used, documented, freely available (http://surfer.nmr.mgh.harvard.edu/) and widely supported by the neuroimaging community. FreeSurfer calculates various morphometry metrics in two processing streams: a surface-based and a volume-based stream (see Textbox 1 and Fig. 1 for an overview of these metrics, their computation, and format). Cortical surface-based reconstruction (left side of Fig. 1) is based on geometric models of the cortical surface and the identification of the borders between certain tissue types (described in detail in Dale et al., 1999). The boundary between the pia mater and cortical gray matter forms the pial surface (medium blue in Fig. 1), while the boundary between cortical gray and white matter represents the white surface (light blue in Fig. 1). The cortex is further modeled as a surface with a mesh of triangles (not depicted in Fig. 1). Each meeting point of triangles (called a vertex) has exact coordinates which allows various non-linear manipulations, like inflation, to perform spatial normalization and group analysis, and to improve visualization. These reconstructions allow for a differentiation of cortical volume, thickness, surface area, mean curvature, and local gyrification patterns. For cortical thickness (turquoise in Fig. 1), FreeSurfer calculates the distance between the pial and white surface (Fischl & Dale, 2000). Cortical volume represents a product of cortical thickness and surface area (gray in Fig. 1). Local gyrification index quantifies the gyrification at each vertex on the surface (yellow in Fig. 1), and is computed in a 3D fashion using a circular region of interest (ROI; 20 to 25 mm) around each vertex (Schaer et al., 2008). Recent studies indicated different genetic, cognitive, and clinical correlates for different cortical morphometry metrics (Nissim et al., 2017; Raznahan et al., 2011; Winkler et al., 2010), emphasizing their independent development.

The volume-based stream (right side of Fig. 1) applies five stages when analyzing T1-weighted raw data (Fischl et al., 2002, 2004) and was developed independently from the surface-based stream. It labels each voxel in cortical and subcortical tissue in a skull-stripped mask of the brain depending on voxel intensity and probability maps. Consequently, the volume of subcortical structures (e.g. nucleus caudatus, thalamus, putamen, globus pallidus, amygdala, and hippocampus), cerebellar gray and white matter, cortical gray matter, and cerebral white matter is calculated (see Fig. 1). FreeSurfer also calculates “estimated total intracranial volume” (eTIV; also called intracranial volume or ICV), using an atlas-representative template and the Atlas Scaling Factor (ASF) which represents “the whole-brain volume expansion (or contraction) required to register each individual to the template” (Buckner et al., 2004, p. 725). For visualization of eTIV, please see Fig. 3 in Buckner et al. (2004, p. 728). This metric may also be used in cross-sectional comparative studies to adjust for individual differences in brain size (see section Statistical analysis). Alternatively, whole brain volume may be used for adjustment. Typically, it is calculated by summing the gray and white matter volumes, excluding the brainstem. Still, whole brain volume may vary depending on researchers’ choice to include non-brain matter such as cerebrospinal fluid, ventricles, and choroid plexus.

Other semi-automated tools exist that analyze several of the aforementioned metrics, for example, AFNI (https://afni.nimh.nih.gov/), Brain Visa (brainvisa.info), Brain Voyager (brainvoyager.com), CARET (brainvis.wustl.edu/wiki/index.php/Main_Page), CAT12 (http://www.neuro.uni-jena.de/cat/), FSL (https://fsl.fmrib.ox.ac.uk/fsl/fslwiki), and MindBoggle (https://mindboggle.info/). Please see Mills and Tamnes (2014) and Popescu et al. (2016) for overviews. Moreover, processing pipelines like the Human Connectome Project (HPC) pipeline (Glasser et al., 2013) combine several tools to facilitate multi-modal neuroimaging analysis, including sMRI, resting-state MRI, task functional MRI, and diffusion MRI.

#### Textbox 1. Brain Morphometry Metrics Given by FreeSurfer (Fischl, 2012).

ATLAS-based Spatial Normalization **Estimated Total Intracranial Volume (eTIV)**

Also called intracranial volume (ICV); may be used for global brain size adjustment when analyzing cortical volume, subcortical volume, and surface area in cross-sectional group comparisons. Reported in mm^3^ or ml.

Surface-based Analysis **Surface area**

Area of the brain surface, spanning two metrics with the same topology:

1. White surface (inner surface area): area of the boundary between white and gray matter. Reported in mm^2^.
2. Pial surface (outer surface area): area of the boundary between gray matter and pia mater. Reported in mm^2^.

**Cortical thickness**

Distance between white surface and pial surface; calculated by finding the closest point on the opposite surface. Reported in mm.

**Cortical gray matter volume**

Also called cortex volume; represents the volume inside the pial surface minus the volume inside the white surface minus tissue inside the ribbon that is not part of the cortex (e.g. hippocampus). Reported in mm^3^ or ml.

**Mean curvature**

Indicator for degree of cortex folding with increased curvature indicating increased or sharper folding; Calculated by the average of two principal curvatures of white matter or pial surface; measured in 1/r, where r is the radius of an inscribed circle. Reported in mm^-1.

**Local gyrification index**

Quantifies the amount of cortex buried within the sulcal folds as compared with the amount of cortex on the outer visible cortex; extensive folding indicates large gyrification index whereas limited folding indicates small gyrification index. Has no unit.

Volume-based Analysis **Subcortical volumes**

Gray matter volume of various segmented subcortical structures (e.g. nucleus caudatus, thalamus, putamen, globus pallidus, amygdala, hippocampus, ventral diencephalon, and substantia nigra). Reported in mm^3^ or ml.

**Cerebellar gray and white matter volume**

Gray and white matter volume of the cerebellum. Reported in mm^3^ or ml.

**Cortical gray matter volume**

Also called cortex volume; sum of all cortical label voxels. Reported in mm^3^ or ml.

**Cerebral white matter volume**

Total volume inside the white surface minus anything that is not white matter, does neither include cerebellar white matter nor brain stem. Reported in mm^3^ or ml.

## Typical and Atypical Structural Brain Development

The objective to track structural brain alterations and understand the underlying mechanisms of neurodevelopmental disorders requires researchers and clinicians to first characterize typical neurodevelopment. Components of cortical and subcortical morphometry develop along different trajectories throughout childhood into adolescence and early adulthood (see e.g. Herting et al., 2018; Tamnes et al., 2017; Vijayakumar et al., 2016). In the next section we will first review typical neurodevelopmental trajectories, followed by a review on alterations in the externalizing disorders ADHD, CD, and ODD. For more details and schematic illustrations of typical neurodevelopmental trajectories for various brain measures, we refer to Mills and Tamnes (2014).

### Typical Structural Brain Development

Mills et al. (2016) identified different trajectories for *whole brain volume* (here a composite measure of white and gray matter including the cerebellum) and *eTIV* (atlas-based spatial normalization procedure using a scaling factor) across four longitudinal samples. While whole brain volume increased across childhood, peaked at age 13 followed by a gradual decrease in adolescence and stabilized in the early twenties, eTIV showed an annual increase of around one percent between late childhood and mid-adolescence, followed by a stabilization in late adolescence (Mills et al., 2016).

*Cortical gray matter volume*, which comprises both cortical thickness and surface area, follows an inverted U-shape trajectory with an increase during early childhood followed by decrease during later childhood and adolescence, and into adulthood (Gilmore et al., 2012; Mills et al., 2016). The decrease of gray matter volume from childhood into adulthood seems to be largely driven by decreases in cortical thickness (Storsve et al., 2014; Tamnes et al., 2017). According to pioneering studies, regions serving primary functions such as the visual and motor cortex show a gray matter peak earlier, while the peak in higher-order association areas such as the prefrontal and temporal cortex appears later (Gogtay et al., 2004; Sowell et al., 2004). However, more recent longitudinal studies did not replicate a peak of gray matter during late childhood or adolescence. Instead, results suggest that gray matter volume is highest before 8 years of age (Mills et al., 2016), decreases across the second decade (Tamnes et al., 2013; Wierenga et al., 2014), and stabilizes in the third decade (Mills et al., 2016). For *cortical thickness*, recent studies reported a monotonic decrease from childhood to early adulthood (Tamnes et al., 2017; Walhovd et al., 2017). Generally, *cortical surface area* showed smaller changes relative to cortical thickness and volume (Tamnes et al., 2017). Increase was reported until about 9 years of age (Wierenga et al., 2014) followed by stability or relatively smaller decrease during adolescence (Amlien et al., 2016; Tamnes et al., 2017; Vijayakumar et al., 2016; Wierenga et al., 2014). Although being one of the most prominent features of the human brain, a unified model on the development and role of *cortical folding* does not yet exist. Originally, cortical shape was seen as a product of underlying patterns of connectivity (see axon tension-based model of convolutional development; Essen, 1997). However, more recent investigations on the development of cortical folding focus on genetic influences (Alexander-Bloch et al., 2020), as well as mechanical (stiffness and elasticity), and cellular mechanisms (Llinares-Benadero & Borrell, 2019). So far, a smaller number of studies investigated the development of curvature and gyrification patterns. These mostly found decreasing gyrification from childhood to adulthood (Mutlu et al., 2013; Raznahan et al., 2011). Concerning *subcortical brain structures*, the basal ganglia (i.e. nucleus caudatus, putamen, and globus pallidus), nucleus accumbens, and cerebellar gray matter showed volume decreases from ages 8 to 22 (Tamnes et al., 2013), while the amygdala and hippocampus appeared to increase with age during adolescence (Durston et al., 2001; Giedd et al., 1996) or showed little to no change (Tamnes et al., 2013). Consistent with these data, a recent study reported slight nonlinear increase for hippocampus and amygdala volume from age 10 to 22 (Herting et al., 2018). In contrast to cortical, subcortical and cerebellar gray matter volumes, which for the most part decrease in adolescence, *white matter volume* has been shown to increase throughout childhood and adolescence, and possibly even further (Mills et al., 2016; Westlye et al., 2010).

Although longitudinal studies are the gold standard and were widely used in recent years to characterize development, please note that contradictory findings exist. These likely stem from specific methodological challenges of longitudinal projects like age range, number of assessments, sample characteristics, image processing techniques, and longitudinal statistical analytic methods. These challenges and best practices have been recently reviewed by Vijayakumar et al. (2018).

### Alterations in the Externalizing Disorders ADHD, CD, and ODD

In contrast to typical development, almost all studies on the neurodevelopmental disorders ADHD, CD, and ODD are cross-sectional, that is, focus on age-independent or age-related differences between patient and control groups, as opposed to differences in development (but see longitudinal studies from Shaw et al., 2012, 2013, 2014). Overall, distinct alterations in neurodevelopmental disorders emerge for cortical and subcortical metrics (see e.g. meta-analysis on ADHD: Hoogman et al., 2017; Nakao et al., 2011 and meta-analysis on CD or ODD: Noordermeer et al., 2016). Please note that results for these neurodevelopmental disorders are biased given high comorbidity rates between ADHD and CD or ODD in most studies, complicating interpretation (Vetter et al., 2020).

For ADHD, reductions have recently been found in eTIV (5%; Vetter et al., 2020), as well as in total brain volume (2.5%; Greven et al., 2015). Concerning *cortical gray matter volume*, a meta-analysis with children and adolescents with CD or ODD revealed reductions in the bilateral insula and the left middle/superior frontal gyrus, possibly indicating compromised hot executive functions (e.g. emotion processing, empathy, and introspection) in CD or ODD (Noordermeer et al., 2016). In ADHD, studies showed decreased (Ambrosino et al., 2017), but also increased (Semrud-Clikeman et al., 2014) volume of the prefrontal cortex. Moreover, delayed *cortical thinning* in prefrontal areas in ADHD has been interpreted as a maturational delay in regions important for cognitive control processes, including attention and motor planning (Shaw et al., 2007). Children and adolescents with CD showed reduced cortical thickness within the parietal lobe, paracentral lobule, precuneus (Hyatt et al., 2012), and superior temporal cortex (Wallace et al., 2014). The thickness of the latter region also correlated with callous-unemotional traits, the core emotional component of psychopathy (Wallace et al., 2014). Concerning *surface area*, a reduced total, frontal, temporal, and parietal area has been found in children and adolescents with ADHD (Noordermeer et al., 2017), as well as a delayed developmental trajectory of the prefrontal cortex surface area (Shaw et al., 2012). For CD or ODD, a reduced prefrontal surface area (Fairchild et al., 2015; Sarkar et al., 2015) or no surface area alterations have been reported (Wallace et al., 2014). Furthermore, Shaw et al. (2012) reported no alterations in *cortical gyrification* for children and adolescents with ADHD from ages 10 to 18, neither in baseline gyrification nor in developmental trajectories. Similarly, no alterations in developmental trajectories for intrinsic curvature and local gyrification index were found for adolescents with ADHD (Forde et al., 2017). For 22 children and adolescents with CD, decreased gyrification has been shown in the right ventromedial prefrontal cortex (Wallace et al., 2014), yet this finding did not survive cluster correction. Moreover, Hyatt et al. (2012) found widespread folding deficits in a similar sample, located mainly in anterior brain regions, including the left anterior insular cortex. In line with the somatic marker hypothesis, alterations in this region may be related to deficits in the formation of subjective feeling and empathy (Medford & Critchley, 2010). Concerning *white matter volume*, no alterations were found neither for ADHD, nor for CD (Greven et al., 2015; Stevens & Haney-Caron, 2012).

For *subcortical brain structures*, a recent mega-analysis spanning 1713 participants with ADHD aged 4 to 63 years found volume reductions in the amygdala, nucleus accumbens, hippocampus, and putamen (Hoogman et al., 2017). Here, the biggest effects emerged in the amygdala, which emphasizes the role of emotional dysregulation in ADHD (Hoogman et al., 2017). In children with CD or ODD, volume reductions in the striatum, amygdala, and hippocampus (Noordermeer et al., 2017; Rogers & Brito, 2016) seem to mirror deficits in emotion processing and decision-making. In sum, there is evidence for alterations in cortical and subcortical structures in children and adolescents with neurodevelopmental disorders such as ADHD and CD or ODD. Affected regions seem to reflect distinct mechanisms underlying the symptomatology of each disorder, that is, attention processes, motor planning, and emotional regulation in ADHD, as well as empathy, introspection, and emotion processing in CD and ODD.

Still, longitudinal studies with large samples are mostly missing, presumably in part due to challenges involved in conducting longitudinal studies with patient groups. However, longitudinal studies are critically needed to probe brain developmental trajectories in neurodevelopmental disorders, both at the group and individual level, as these may yield different results than case-control studies testing for age-effects (see e.g. discussion about limitations with cross-sectional designs in Kraemer et al., 2000). Longitudinal studies are needed to inform us about the ontogeny of structural brain alterations in neurodevelopmental disorders, and ultimately to track developmental trajectories in individuals with psychopathological risk factors or a clinical diagnosis.

Finally, several findings remain inconclusive, especially when trying to unravel the unique role of the different brain structure metrics in atypical neuromaturation. Divergent findings might arise, among other reasons, from methodological heterogeneity in image acquisition, image data processing, and statistical analysis. Hence, we will now present guidelines with the goal to help clinical researchers improve their practices and ultimately to gain more robust, cross-study comparable results for the field. In Textbox 2 we include a non-exhaustive summary of important considerations in study design and guidelines for image acquisition and analysis, quality control, and statistical analysis, which apply to all studies in clinical developmental structural neuroimaging research.

#### Textbox 2. Guidelines for Reporting Methodological Details in Clinical Developmental sMRI Research

Study Design ✓ Consider generalizability of sample during recruitment and report details (i.e. age, IQ, socioeconomic status, pubertal status, and ethnicity)

✓ Report physical and psychiatric comorbidities

✓ For the patient group:

-
  - Report characteristics for (previous) medication and therapy, age of onset and duration of illness for neurodevelopmental disorder
-
  - Report diagnostic procedure
    -
      - confirmed by whom and how (e.g. through questionnaires and clinical interviews by registered psychologist or study staff)
    -
      - cut-offs to define clinical psychopathology
    -
      - specify subtypes and severity

✓ Match groups according to e.g. sex and age; provide information on matching strategy

✓ Provide information on missing data (e.g. questionnaires, medication status, IQ)

##### Image Acquisition

✓ Implement and report protocols to improve comfort and thus reduce motion

✓ Report on participants’ motion

✓ Consider acquisition techniques (e.g. fMRI as proxy, PROMO) for motion-correction

✓ Avoid change in scanner hardware, sequences and protocols across sites and participants

-
  - If not possible, account for differences in all analyses

##### Image Processing and Quality Control

✓ Employ same software (and version) across all participants within a study and report details

✓ Give preference to software that covers brain metrics and regions of interest, chose these a priori based on literature and hypotheses, and report details

✓ Report on quality control procedure

-
  - Inspection of the quality of raw and processed images with specification of exclusion criteria
-
  - Tools or algorithms used during quality control procedure
-
  - Manual changes/trouble shooting techniques

##### Statistical Analysis

✓ Consider e.g. sex, age, and global brain size as covariates

-
  - If correcting for global brain size:
    -
      - Report the brain metric and correction method used
    -
      - Report results from both raw and corrected regional brain measures
    -
      - Take into account relationships between sex, age, and global brain size

✓ Appropriately account for the multivariate nature of the data by:

-
  - Correcting for multiple comparisons applying suggested thresholds for different brain metrics
-
  - Conducting multivariate analyses

##### Conclusions

✓ Interpret findings within the bounds of the analytic technique and in line with the statistical analyses performed (e.g. as relative differences if results were adjusted for global brain size)

## Steps to Successful Clinical Developmental Structural Neuroimaging Research

### Study Design

Decisions on the study design depend on the goal of the clinical developmental sMRI study (Greene et al., 2016). If one seeks to understand underlying mechanisms of a disorder, a “clean” sample, that is, participants with symptoms of the neurodevelopmental disorder of interest who do not receive medication or treatment, is preferable (Greene et al., 2016). However, this is seldom feasible as most patients with neurodevelopmental disorders receive some kind of treatment. In addition, samples that are more naturalistic allow for investigation of treatment effects (e.g. medication). Further, studies investigating neurodevelopmental disorders are often not capable of including large enough patient groups to compare effects of important, possibly confounding variables such as disorder subtypes, comorbidities, medication, and other treatments. Decisions made at this stage regarding recruitment and study focus will substantially affect the capability to conduct statistical analyses and reliably interpret results.

Many challenges of study design in clinical neurodevelopmental sMRI studies have already been discussed in detail elsewhere, spanning participant inclusion, sample composition (sample size, high versus low-functioning participants, subtypes, and comorbidities), medication, and other treatment history, as well as considerations for control groups (Bednarz & Kana, 2018; Greene et al., 2016). Therefore, we kindly refer to these publications. For discussion on statistical power and selective, small or non-representative samples, please refer to Klapwijk et al. (2019a, 2019b). We will further present considerations for image acquisition, quality control and image processing.

## Image Acquisition and Immediate Quality Control

Most studies including MRI examinations (i.e. functional task-based/resting-state MRI or diffusion MRI) also run a T1-weighted sequence by default to check for neuroanatomical anomalies and to coregister data. The FreeSurfer Wiki (https://surfer.nmr.mgh.harvard.edu/fswiki) recommends T1-weighted sequence acquisition protocols for use with the FreeSurfer processing pipeline. Contrary to functional MRI, which aims to monitor brain activity, the measurement of brain morphometry is not timing sensitive during one MRI session. In addition, the acquisition time of typical sequences (≈1 mm isotropic) is relatively short (5-10 minutes) and users may inspect quality of T1-weighted images immediately after scan completion. Hence, if data quality was not satisfactory, such a short T1-weighted sequence can often be repeated (see also Backhausen et al., 2016) and a time buffer of 15 minutes included in the MRI session schedule may help reduce data loss in challenging samples like children and adolescents with neurodevelopmental disorders. Therefore, personnel present at the scanner should learn about different types of artifacts (mostly technical and motion artifacts) and their impact on the data to be able to identify compromised data quality and decide on the necessity to rescan. Technical artifacts include head coverage, radiofrequency noise, signal inhomogeneity, and susceptibility (Costa et al., 2009; Reuter et al., 2015; Wood & Henkelman, 1985). Motion artifacts result from the participant swallowing, blinking, chewing, turning, fidgeting or repositioning a limb (Bellon et al., 1986; Zaitsev et al., 2015). Younger age groups tend to produce more motion artifacts (Blumenthal et al., 2002; Satterthwaite et al., 2013; Van Dijk et al., 2012; Yuan et al., 2009). Moreover, images of children and adolescents with neurodevelopmental disorders might be particularly prone to motion artifacts due to the symptoms themselves (see for ADHD: Backhausen et al., 2016; Rauch, 2005; Vetter et al., 2020, and for CD: Huebner et al., 2008). Our group recently demonstrated that good quality of structural data can be acquired in clinical developmental samples such as ADHD and CD or ODD when applying a systematic and detailed workflow (Backhausen et al., 2016). A further important procedure to reduce movement is to ensure compliance by age-appropriate instructions with film clips, a playful approach like the “statue game”, or by using a mock scanner (Raschle et al., 2009), allowing the participants to understand and practice the importance of holding still. These preparations can even be individualized in the form of personalized familiarization strategies and rewards to enable children and adolescents with neurodevelopmental disorders to better tolerate the MRI scanner environment and ensure high quality images (Pua et al., 2020).

Moreover, prospective motion correction (PMC) techniques exist which track the participants’s head motion during the scan (e.g. through volumetric navigators) and modify pulse sequences to correct for participant motion (Tisdall et al., 2016). PMC techniques specific to the three main scanner manufacturers include PROMO for GE (White et al., 2010) and motion tracking systems for Siemens (Zaitsev et al., 2006) and Philips (Ooi et al., 2009). To evaluate the costs and benefits of PMC, a recent study compared reliability and quality of structural imaging in traditional Magnetization-Prepared Rapid Gradient-Echo (MPRAGE) versus MPRAGE with PMC in a developmental data set (Ai et al., 2020). They reported higher intra-sequence reliability but poorer quality metrics in scans with PMC compared to the traditional MPRAGE and noted that scans with PMC were robust but not fully resistant to high head motion (Ai et al., 2020). In conclusion, Ai et al. (2020) recommend the use for hyperkinetic populations when increased motion can be expected (i.e. children, patients with neurodevelopmental disorders).

Still, from our experience, data quality can and should be evaluated at different stages in an sMRI study. First, check acquired images at the scanner console after running the structural scan to allow for re-scan if needed and possible. Second, visually rate data quality of acquired raw T1-weighted data sets according to a standard rating system or using an automated method, and third, check data sets after they have been processed, again, either visually or using an automated method (see below and Backhausen et al. (2016) for a detailed workflow suggestion).

Another important concern of image acquisition is the reliability, that is, overall consistency of MRI-derived output metrics. Reliability might vary across MRI scanners, field-strength and head-coils (Heinen et al., 2016). As higher field strength improves signal-to-noise ratio and spatial resolution (Tijssen et al., 2009), 3 Tesla MRI scanners are preferable as compared to 1.5 Tesla MRI scanners. More recently, ultrahigh-field 7 Tesla MRI has been utilized given it provides greater signal-to-noise ratio, contrast-to-noise ratio, and increased spatial resolution as compared to lower magnetic fields (Barisano et al., 2018). Although 7 Tesla MRI scanners became FDA approved for clinical use in October 2017 (Barisano et al., 2018), and a few studies, including the Human Connectome Lifespan (https://www.humanconnectome.org/study-hcp-lifespan-pilot/phase1b-pilot-parameters), have successfully imaged children 8 years and older on 7 Tesla MRIs (albeit not structural MRI but rather to collect resting-state and diffusion MRI), 3 Tesla MRI has been more widely used world-wide. Moreover, 7 Telsa human brain imaging still has some limitations and faces technical challenges, including increased specific absorption rate (SAR) and increased sensitivity of inhomogeneity and motion artifacts (Barisano et al., 2018). Participant protections are also unique given potential increases in nausea, claustrophobia or dizziness. As advances in magnetic resonance technology tackle these issues, mainstream imaging with 3 Tesla may eventually be replaced by 7 Tesla protocols. Regardless of field strength, MRI technologists or physicists should regularly perform data quality checks (e.g. signal-to-noise) to ensure stable machine characteristics and performance. It is imperative that all participants are scanned using the same hardware, software, and MRI sequence to avoid increased variability or even systematic bias. If unavoidable, these effects should be estimated and statistically accounted for (Lee et al., 2019), either by including site, scanner or sequence as a covariate in statistical analysis or by applying tools developed for multi-site harmonization. The recent algorithm ComBat removes unwanted non-biological variability associated with different properties of MRI scanners from cortical thickness data in cross-sectional (Fortin et al., 2018) and longitudinal studies (Beer et al., 2020), which may increase power and reproducibility of subsequent statistical analysis.

## Image Processing

The choice of an sMRI data processing tool depends on the research question and regions or metrics of interest. Since tools differ with regard to reconstruction algorithms and brain atlases, it is important to learn about the specific nomenclature of brain regions available for ROI-based analysis. Importantly, all tools, although appearing to offer automatic processing, require thorough quality control and other “manual” decisions. We will thus term them “semi-automatic”.

**Fig. 2:**
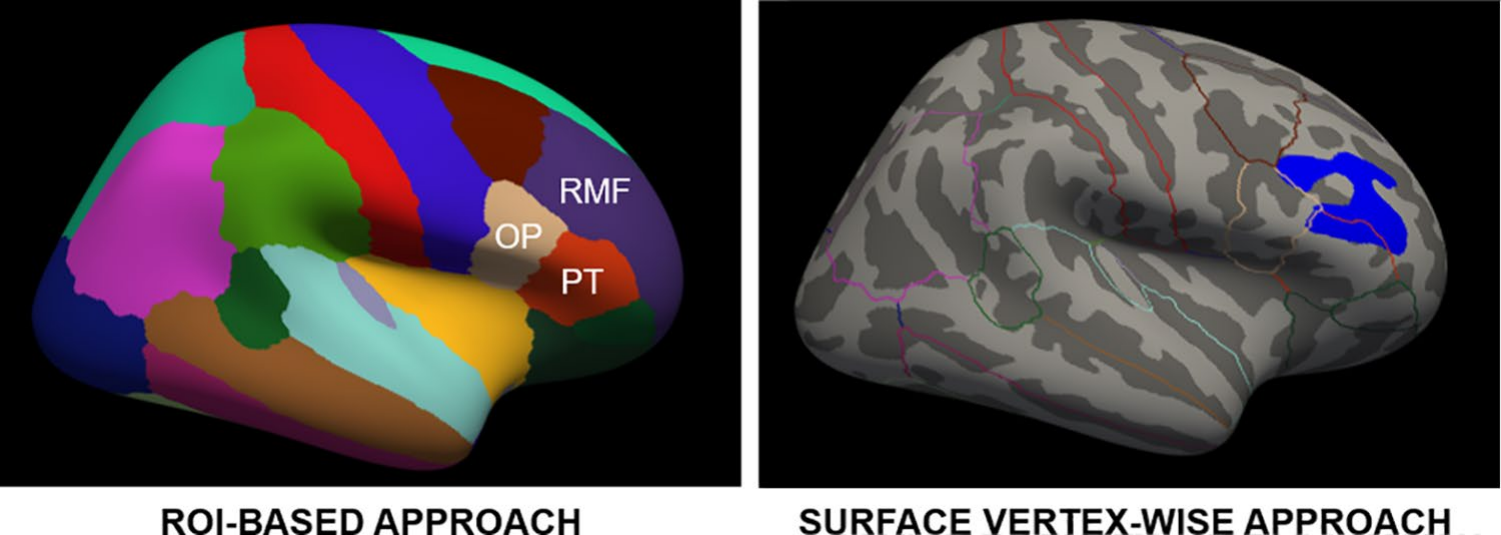
ROI-based versus surface vertex-wise approach as implemented in FreeSurfer. ROI-based approach depicting the lateral part of the right hemisphere with Desikan-Kiliany atlas regions (left) and surface vertex-wise approach (right) for statistical analysis of clinical developmental sMRI data. The hemisphere is inflated for a better view of gyri and sulci. The blue area on the right picture highlights a region with significant differences in cortical thickness between two groups, which falls partly into pars triangularis and rostral middle frontal cortex as indicated by Desikan-Kiliany atlas region outlines. Using the ROI-based approach this difference may or may not contribute to significant cortical thickness differences in the rostral middle frontal cortex, pars triangularis, or pars opercularis without the possibility of localizing the area more precisely. OP = pars opercularis; RMF = rostral middle frontal cortex; PT = pars triangularis

As previously outlined in detail, FreeSurfer (Fischl, 2012) computes volume-based and surface-based metrics. Using nonlinear transformations, cortical measurements are spatially re-sampled onto a standard surface-based template (fsaverage), which represents an average brain (Sabuncu et al., 2014). By default, FreeSurfer provides outputs of these estimates using the option of two standard atlases: the Destrieux atlas (Destrieux et al., 2010) and the Desikan-Killiany atlas (Desikan et al., 2006; see Fig. 2). In adults, the test-retest reliability tends to be higher when using the Desikan-Kiliany compared to the Destrieux atlas (Iscan et al., 2015); albeit to our knowledge a similar comparison has not yet been performed in children and adolescents.

For more than one data set per participant, researchers and clinicians should use the FreeSurfer longitudinal stream (Reuter et al., 2010). This pipeline includes surface- and volume-based image processing in three steps: 1) CROSS: processing of every data set cross-sectionally, 2) BASE: template creation, and 3) LONG: reanalyzing cross-sectional data sets with information from the BASE template. This process improves robustness compared to conventional cross-sectional analysis pipelines (Jovicich et al., 2013).

To sum up, researchers and clinicians need to keep in mind that output metrics and nomenclatures vary between image processing tools and their brain atlases, complicating interpretation of results. When comparing results of structural alterations in clinical developmental samples, it is thus crucial to consider the size and location of brain regions used for ROI-based analysis and how they compare to similar regions from other brain atlases implemented in different semi-automated processing tools. Moreover, for all neuroimaging studies, but perhaps especially for developmental clinical sMRI, the next important step is to check data to ensure good data quality for valid results. We will thus present considerations on quality control of both raw and processed data in the next passage.

## Quality Control After Image Acquisition

Bad image quality can lead to poor image reconstruction with semi-automated tools and, importantly, to underestimation of gray matter volumes and cortical thickness (Backhausen et al., 2016; Blumenthal et al., 2002; Reuter et al., 2015). One study also demonstrated the impact of quality control procedures on developmental trajectories, as quadratic trajectories of cortical thickness across ages 5 – 22 years could no longer be identified when applying a more stringent quality control as opposed to the standard form (Ducharme et al., 2016). Hence, stringent quality control procedures are of critical importance in clinical developmental sMRI studies to prevent biased results on case-control differences or on developmental trajectories.

Conventional quality control procedures include visual inspection of raw or processed T1-weighted images, without knowing patient versus control participants to reduce inherent bias. First, evaluating them according to a rating system including several quality markers (e.g. blurring, ringing, signal-to-noise ratio of the gray and white matter borders and subcortical structures, see rating system proposed by Backhausen et al., 2016) and then sorting scans into categories (e.g. as “pass”, “warn” or “fail” by Backhausen et al., 2016; Reuter et al., 2015). Those categorized as “fail” should then be excluded from further data analysis. Implementing visual quality control based on rating systems like these can be challenging as it usually requires experienced raters and is excessively time consuming, especially for large data sets. Yet, the need of increased transparency and detailed reports of quality control protocols has to be emphasized, as to this point several previous studies did not include any description of quality control (Vijayakumar et al., 2018). Arguably, both inter-rater as well as intra-rater variability of visual quality control is generally high but may still lead to inclusion of poor quality scans and exclusion of scans of usable quality (Klapwijk et al., 2019a, 2019b). In an attempt to expand the conventional visual quality control, we present a non-exhaustive selection of recently developed techniques in Table 1. They are evaluated concerning quality control input measures, visual quality control and classifier categories, techniques, quality control outputs, and performance.

**Table 1:** Quality control approaches for sMRI data

Method	QC input metrics	Visual QC/ classifier categories	Technique	QC output	Performance
FD (Savalia et al., 2017)	FD from functional MRI scan of the same session as proxy for motion in T1-weighted images	Three categories: pass, warn, fail	Flagging procedure; combining visual QC and estimates of head motion from functional MRI scans	FD estimates and visual QC ratings	FD estimates complement visual QC rating
Euler number (Rosen et al., 2018)	Euler number outputted by FreeSurfer	Three categories: 0 (gross artifacts/fail), 1 (some artifacts but usable), 2 (no artifacts)	/	Euler number, no specific recommendations	Euler number as most accurate quality measure/highest correlation with visual QC
MRI-QC (Esteban et al., 2017)	Raw T1-weighted images, 64 IQMs per input image	Binary classifier: include, exclude	random forests classifier trained on a publicly available, multi-site data set (17 sites, *N* = 1102)	individual anatomical reports (calculated IQMs and metadata in the summary, as well as a series of image mosaics and plots designed for the visual assessment of images)	Intra-site prediction: high accuracy; Unseen site prediction: leaves space for improvement (76 % ± 13 % accuracy)
Qoala-T (Klapwijk et al., 2019a, 2019b)	Metrics form the FreeSurfer output files aseg.txt, aparc_area.txt and aparc_thickness.txt (all for both hemispheres) including the variable surface holes	Four categories: 1 (excellent), 2 (good), 3 (poor), 4 (failed)	supervised-learning model, random forests classifier trained on the BrainTime data	Qoala-T score (ranging from 0 to 100), recommendation whether to visually check and whether to include or exclude each data set from further analyses	Intra-site prediction: high accuracy (mean AUC = 0.98); Unseen site prediction: similar accuracy (mean AUC = 0.95)

*AUC* area under the curve, *QC* quality control, *FD* frame-by-frame displacements, *IQM* image quality metrics, *MRI* magnetic resonance imaging

The first approach shown in Table 1 uses the Euler number from the standard FreeSurfer output as a proxy for cortical reconstruction quality. It represents the number of topological defects which may occur during the surface retesselation step when structural data sets are reconstructed by the FreeSurfer pipeline. An Euler number of 2 (equal to that of a sphere) indicates a smooth cortical reconstruction. However, as stated by Fischl, Liu, & Dale (2001), two types of defects may occur during the reconstruction process, termed “holes” and “handles”. Holes consist of small perforations in planar sheets of white matter and handles are bridges between nonadjacent points in the cortex. Each defect of either type reduces the Euler number and FreeSurfer seeks to maximize the Euler characteristic to a value of 2 in subsequent topology correction steps. This quality measure was recently found to be consistently correlated with visual quality control ratings across samples, being able to identify images scored “unusable” by human raters with a high degree of accuracy (area under the curve (AUC): 0.98–0.99; Rosen et al., 2018). Rosen et al. (2018) reported z-scores for Euler numbers, and advised researchers to determine classification thresholds for each specific data set. This approach was followed by Yu et al. (2018) who identified three cases as outliers according to the averaged Euler number across the left and right hemispheres and thus excluded them from further analyses. Here, Yu et al. (2018) set the threshold for outliers to averaged Euler numbers < 3.29 standard deviations below the sample mean.

Furthermore, the frame-by-frame displacement (FD) approach uses head movement parameters of functional MRI scanning (average FD calculated for each individual; Power et al., 2014) during the same scan session as a proxy for movement during the structural scan (Savalia et al., 2017). As FD is derived from functional scan output it may be seen as an objective quantitative quality measure in comparison to qualitative visual quality control rating, which is arguably subjective. It is attempted to hereby identify and reduce potential motion-induced bias. Individuals showing excessive head motion during the functional scan combined with bad visual quality control ratings on the structural scans themselves can be “flagged” and subsequently removed from further statistical analysis. Nevertheless, measures derived from functional scans remain imprecise estimations as the severity of participants’ movement may vary across the scanning session.

Using automated machine-learning algorithms, the MRI Quality Control tool (MRIQC; Esteban et al., 2017) extracts 64 image quality metrics (IQM) from raw T1-weighted images, including measurements of variability and specific artifacts in the images, and gives a recommendation whether to include or exclude each image. The tool also provides individual anatomical reports including calculated IQM and metadata, as well as a series of image mosaics and plots designed to assist visual assessment of structural images. MRIQC can be accessed freely online via a web interface using the OpenNeuro.org portal. Moreover, the source code is publicly accessible through GitHub (https://github.com/poldracklab/mriqc) providing a maximum of transparency.

Moreover, the supervised-learning model Qoala-T tool (Klapwijk et al., 2019a, 2019b) predicts manual quality control ratings from FreeSurfer processed metrics including the Euler number (namely “Surface holes” in the FreeSurfer output files), as well as subcortical volumes, cortical thickness, and cortical surface estimates. Researchers can choose either to predict scan quality by using the BrainTime data model provided by Qoala-T or to train the algorithm using ratings of their own data set. Qoala-T scores ranging from 0 to 100 are provided for every individual scan (scores smaller than 50 are advised to be excluded from further analyses). Still, as the algorithm had been originally trained based on subjective visual quality control ratings, the authors recommend to visually check scans with a score between 30 and 70 as misclassified scans are more likely to fall within these boundaries (Klapwijk et al., 2019a, 2019b). The use of the Qoala-T tool could greatly reduce the time needed for quality control as only a part of the data set has to be visually checked. More importantly, this procedure could further help to reduce variability related to visual quality control, thereby benefiting the comparability of data quality between studies (Klapwijk et al., 2019a, 2019b). Qoala-T scores could even be used as a covariate for head motion, that is, motion artifacts in statistical analysis. In sum, these tools provide researchers with a good overview of the quality of their data set and assist decisions about including or excluding data sets.

After good data quality has been insured, more challenges await when statistically testing for brain structure differences in neurodevelopmental disorders. We will address challenges concerning ROI-based versus vertex-wise analysis, covariates, multiple comparison correction, and generalization of results in the next section.

## Statistical Analysis

Statistical analysis affects interpretation of results and may potentially bias decisions concerning diagnosis and treatment of a disorder. For a detailed review on statistical inferences and pitfalls in neuroimaging, see Hupé (2015).

FreeSurfer offers two possibilities to statistically analyze group comparisons and correlations with other variables of interest, namely the *ROI-based and surface vertex-wise approach*. ROI-based includes analyzing the subcortical and cortical volumes, cortical thickness, surface area, and local gyrification values according to pre-defined atlases (Destrieux or Desikan-Kiliany; see section Image processing). ROIs should be derived a priori based on literature review and with specific hypotheses, as an excessive number of ROIs may lead to problems during multiple comparisons correction. By contrast, vertex-wise group analysis fits a between-subject general linear model at each particular surface vertex to compare values of cortical thickness, surface area, volume or local gyrification. Statistical maps are then overlaid on a template brain as a surface map representing contrast estimates in different colors. This approach may be useful in explorative studies without a priori hypotheses on affected brain regions. Note that FreeSurfer users can conduct a ROI-based analysis with segmented subcortical and parcellated cortical metrics, while the vertex-wise analysis is only available for cortical metrics. Choosing one or the other approach depends on the effects of interest. If regions with significant group differences follow gyral borders defined in a ROI-based atlas, using these pre-defined parcellations may increase statistical power. On the downside, effects in smaller or more specific regions might not be detected as values are averaged within one parcellated ROI (see Fig. 2). Finally, both methods may be used in one study to complement each other.

Furthermore, researchers and clinicians need to consider several *covariates* during statistical analysis of clinical neurodevelopmental sMRI data as they may confound between-subject comparisons. These closely intertwined variables include age, sex, and different global brain size measures. Researchers and clinicians should thoroughly look into the methods and metrics to use for correction and take them into account when interpreting results. The participants’ age matters especially during periods of dynamic neurodevelopment like childhood and puberty, as regional brain metrics change with time (see section Typical structural brain development). Therefore, researchers and clinicians should examine scaling of regional metrics of interest with age within each group and interpret group differences with respect to the metrics’ underlying developmental trajectory. Concerning sex differences in structural brain development, boys have larger head and brain sizes (De Bellis et al., 2001; Paus et al., 2017; Sowell et al., 2002) as compared to girls. Moreover, subtle sex differences have been reported for cortical thinning during adolescence (higher rate of cortical thinning in females in right temporal regions and the left temporoparietal junction; Mutlu et al., 2013). As sex differences during neurodevelopment may be associated with a different age of onset and clinical presentation of neurodevelopmental disorders, sex should always be considered in statistical analysis, for example, by including it as a covariate.

Moreover, accounting for global brain size or not is a particularly important issue in cross-sectional comparative studies, since differences between groups may be driven by interindividual global brain size differences and may not be inherent to the regional metric of interest. In longitudinal developmental studies, this issue is more complex, as global brain size measures may themselves change with age in children and adolescents. The decision whether or not to correct for global brain size (O’Brien et al., 2011) and which metric and method to use for this correction (Mills et al., 2016) may affect both results and their interpretation. As already discussed (section Typical and atypical structural brain development), estimations for global brain size differed significantly in past sMRI research, including, for example, cerebral volume (De Bellis et al., 2001), total brain volume (Sowell et al., 2007), whole brain volume, and eTIV (Mills et al., 2016).

Moreover, researchers and clinicians may use three main adjustment methods to correct regional metrics for these global sizes: proportion, analysis of covariance, and residual approach (although the last is rarely used anymore as it may be difficult to interpret; O’Brien et al., 2011). In the proportional method, regional brain metrics of interest are divided by global brain size, leaving a proportional value for further analyses. In group comparisons, this implies the assumption of an identical linear relationship of each brain region and total eTIV or whole brain volume between the groups. If these conditions are not met, calculating a proportion may introduce a bias. The analysis of covariance method accounts for shared variance with global brain size by regression statistics through the inclusion of global brain size as a covariate into the analysis. Due to its simpler implementation the analysis of covariance method is preferred, especially in vertex-wise analysis, as the proportional method would imply adjusting each cortical vertex prior to statistical analysis (Vijayakumar et al., 2018). Notably, these variables may affect results of corrected regional brain metrics differently, as developmental trajectories of cortical gray matter volume from childhood to adulthood differed depending on the adjustment method and metric for brain size (eTIV or whole brain volume; Mills et al., 2016). Furthermore, eTIV and whole brain volume have both been found to account for sex differences in gray matter development during adolescence using the proportional adjustment method, yet only whole brain volume did so when applying the analysis of covariance adjustment method (Mills et al., 2016). Hence, the relationship between brain size and sex should be carefully investigated when including these two covariates in statistical analysis. For a further discussion of the assumption of linear scaling between regional and global brain size, please also see Vijayakumar et al. (2018).

Importantly, an adjustment for global brain sizes is not always necessary. ETIV should be included as a covariate only when analyzing metrics that scale with global brain size, including subcortical volume, cortical volume, and surface area (see FreeSurfer Wiki; https://surfer.nmr.mgh.harvard.edu/fswiki/eTIV). As cortical thickness is thought to be independent of global brain size, eTIV should not be considered and the majority of research on cortical thickness chose not to control for global brain size (Vijayakumar et al., 2018). Correspondingly, Barnes et al. (2010) propose to adjust for age, sex, and global brain size when analyzing gray matter volume and an adjustment for age and sex when analyzing cortical thickness. Taken together, researchers and clinicians are strongly advised to present results for both raw and corrected brain metrics, report metrics and methods used for correction in detail, and discuss how correction may affect interpretation of results.

A big challenge, especially in vertex-wise analysis, is controlling the false positive rate (FPR) of results. The FPR represents the rate of type-I errors (assuming a group difference when in truth there is none), which should be arbitrarily but conventionally less than 5 % (Benjamin et al., 2018). In clinical developmental structural neuroimaging this is aggravated by the tens of thousands single measurements (vertices) in an image of the brain, where a large number of hypotheses are tested simultaneously. For a summary of approaches to this *multiple comparisons* problem, see Bender and Lange (2001) on when and how to adjust for multiple testing and Greve and Fischl (2018) on specifics in surface-based analysis.

Considering vertex-wise group comparisons in FreeSurfer, parametric gaussian-based Monte Carlo (MC) simulation is used by default to compute the FPR. MC simulation extracts sets of contiguous vertices (clusters) after smoothing and thresholding white noise (cluster forming threshold; CFT) in many iterations, usually 10,000. Subsequently, this algorithm computes the p-value of the clusters in the real data (Greve & Fischl, 2018). This parametric approach relies on gaussian spatial smoothness of data and a gaussian distributed underlying noise, which is not always met in neuroimaging data (Eklund et al., 2016). Non-gaussian permutation offers an alternative approach to multiple comparison correction and was previously found to adequately control FPRs (Winkler et al., 2014). Calculations of both MC simulation and permutation depend on the smoothing kernel (2, 4, 6, 8, 10, or 12 mm full-width/ half-max; FWHM) and CFT (0.05, 0.01, 0.005, or 0.001). In a study testing the performance of these two approaches in vertex-wise analysis of cortical thickness, surface area, and volume, Greve and Fischl (2018) suggested the following when applying MC simulation:

- thickness or volume: CFT ≤ 0.001 and any smoothness level OR CFT ≤ 0.005 with smoothness level FWHM > 10 mm;
- surface area: CFT ≤ 0.001 and smoothness level FWHM > 10 mm

Still, often no vertices survive with such stringent CFTs, greatly reducing power. Permutation allows less stringent CFTs as it adequately controls FPR with all combinations of CFT and smoothness levels. However, permutation comes with some disadvantages including more complicated set up, high computation time, and it requires the data to be exchangeable across participants, a topic discussed in detail by Winkler et al. (2014). Both MC simulation and permutation are available within FreeSurfer statistical analysis using the mri_glmfit-sim script.

For ROI-based analysis, false discovery rate (FDR) is frequently used to handle the multiple-testing problem (Benjamini & Hochberg, 1995). This method can be easily applied with tools like the False Discovery Rate Online Calculator (https://tools.carbocation.com/FDR). Similarly, researchers and clinicians may apply Bonferroni adjustment procedures (Bland & Altman, 1995). The original Bonferroni method is fairly simple but at the same time tends to have low power and should be used for a small number of tests (Bender & Lange, 2001). The Bonferroni method and some improvements like the more powerful Holm method (Aickin & Gensler, 1996), and correction for correlated outcome variables are implemented and easy to apply (e.g. via Simple Interactive Statistical Analysis; https://www.quantitativeskills.com/sisa/calculations/bonhlp.htm). As a weakness of these procedures, the interpretation of a finding depends on the number of other ROIs going into the FDR or Bonferroni correction. Hence, excessive numbers of ROIs reduce the probability of any significant result and truly important differences may be deemed non-significant. In any case, researchers and clinicians should include and transparently report steps taken to correct for multiple comparisons, and discuss possible interpretations of each result to ease comparability between studies.

Results on altered brain structure in neurodevelopmental disorders, even when statistically significant and corrected for multiple comparisons, often need to be interpreted with caution due to small sample sizes and redundant samples when the same participants are utilized in several reports (Anderson & Kiehl, 2012). As mentioned earlier, recruiting enough children or adolescents with neurodevelopmental disorders who fulfill inclusion criteria is often difficult for smaller laboratories. Smaller samples are usually more manageable, and researchers are able to thoroughly check for exclusion criteria in each participant. Concerning the goal to obtain results from clean samples, this is beneficial. Yet, small samples have been shown to more likely yield false significant findings and produce type-I errors with relatively large effect sizes (Ingre, 2013). Larger sample sizes allow for a better estimation of effect sizes (Ingre, 2013), which are usually quite small in sMRI research.

One way to increase the value of small clinical developmental samples is to work together across multiple centers to increase total sample sizes. Researchers and clinicians can pursue this goal via collaborations in multi-center studies. Another option is to take part in global alliances like the ENIGMA (Enhancing NeuroImaging Genetics through Meta-Analysis) consortium, where over 50 diverse working groups participate in post-hoc data pooling and analyses (Thompson et al., 2020). These efforts accounted for the largest sMRI studies to date in several neurodevelopmental disorders (e.g. Hoogman et al., 2017 for ADHD). In doing so, large scale study findings from the ENIGMA-ADHD working group with 36 cohorts from around the world indicated significant cortical thickness reductions in the fusiform, parahippocampal, and precentral gyrus, as well as the temporal pole in 1018 children with ADHD compared to 1048 controls (Hoogman et al., 2019). Likewise, surface area reductions were found with the largest effect size *d* being - 0.21 for total surface area (Hoogman et al., 2019). Importantly, such collaborative approaches heavily rely on transparent standardized procedures (study protocol, hardware and sequences used for acquiring sMRI data, quality control procedure, processing tools, statistical analysis methods etc.). These measures are vital to enable replication studies and to include studies in meta-analyses to combine several smaller studies on the same research questions. To further promote collaboration, the Brain Imaging Data Structure (BIDS) has been developed to organize and describe neuroimaging and behavioral data sets (i.e. file naming and folder organization) prior to image processing to facilitate automatic pipelines and quality control protocols in shared data (Gorgolewski et al., 2016).

Please note that all aforementioned statistical analysis approaches fall under easier implemented univariate methods which assume that differences between groups can be observed in isolated ROIs or vertices but ignore relationships between them (Davis & Poldrack, 2013). Additional multivariate methods are also valuable as they allow for more complex analyses concerning cortical networks like identifying cortical regions that co-vary together (Alexander-Bloch et al., 2013) using, for example, nonnegative matrix factorization (Ball, Beare, and Seal 2019) or machine learning (Peng et al., 2013).

To sum up, it is highly advised to follow guidelines for reporting methodological details in clinical developmental sMRI research. An overview of such guidelines was presented by Vijayakumar et al. (2018) spanning sample, acquisition, processing, analysis, and conclusion. As the authors formulated the guidelines specifically for longitudinal sMRI studies investigating typical brain development, we modified them to apply to clinical developmental sMRI (see Textbox 2). They represent comprehensive guidance when implementing transparency of practices in order to reach accurate understanding of neurodevelopmental disorders.

## Conclusion

In conclusion, we have discussed several important methodological challenges of clinical developmental sMRI research and provided step-by-step hands-on guidelines how to approach them in the order of an sMRI study: study design, image acquisition, image processing, quality control at different stages, and statistical analysis and interpretation. Variation in these approaches at each step may have contributed to differing results and interpretations of typical neuromaturation from childhood to adolescence, and of alterations in these processes in neurodevelopmental disorders. Further research should seek for three things: 1) adopt greater transparency of practice and rationales for decisions on study design, image acquisition, image processing, and statistical analysis, 2) conduct analyses and report findings for each brain morphometry metric to achieve a complete picture of brain structure and maturation and possible alterations, 3) empirically examine the effects of varying methods on results, in order to promote the most robust results possible. Adoption of these criteria will help to ensure findings do not only apply to specific samples or methods, but are robust enough to ultimately promote a solid and accurate understanding of neurodevelopmental disorders.
